# Operative Complications and Outcomes Comparing Small and Large Uterine Weight in Case of Laparoscopic Hysterectomy for a Benign Indication

**DOI:** 10.3389/fsurg.2021.755781

**Published:** 2021-10-05

**Authors:** Shahzia Lambat Emery, Michel Boulvain, Patrick Petignat, Jean Dubuisson

**Affiliations:** ^1^Department of Pediatrics, Gynecology and Obstetrics, Geneva University Hospitals and University of Geneva, Geneva, Switzerland; ^2^University of Geneva, Geneva, Switzerland

**Keywords:** laparoscopic hysterectomy (LH), operative complications, operative outcomes, morcellation of the uterus, uterine weight

## Abstract

**Study Objective:** This study was performed to evaluate the association between uterine weight and operative outcomes in women undergoing laparoscopic hysterectomy for a benign indication.

**Methods:** This is a secondary analysis of a randomized trial with data collected prospectively and retrospectively. The data of 159 women undergoing laparoscopic hysterectomy for a benign indication were analyzed. Women were divided in two groups according to the postoperative uterine weight: small uterus group (<250 grams) and large uterus group (≥250 grams). Operative complications were compared between the two groups. Operative outcomes (need for uterine morcellation, operative duration, estimated blood loss), postoperative pain, and hospital length of stay were also analyzed.

**Main Results:** Operative complications were not significantly different between the two groups (37% in the large uterus group versus 41% in the small uterus group). Operative outcomes showed a significantly increased use of uterine morcellation in the large uterus group (61% in the large uterus group versus 10% in the small uterus group). The operative duration was 150 min in the small uterus group and 176 min in the large uterus group, which corresponds to an increase of 17% in the large uterus group. The mean pain score on the day of surgery was identical in both groups (VAS pain score 5), but significantly in favor of the large uterus group on day 1 postoperatively (VAS pain score 4 in the small uterus group and 3 in the large uterus group). There was no statistical difference between groups in the mean hospital stay (62 ± 37 hours in the small uterus group versus 54 ± 21 hours in the large uterus group). In terms of surgical indication, the small uterus group comprised more patients with endometriosis/adenomyosis (36%) and the large uterus group more patients with leiomyoma (93%).

**Conclusion:** The results from this study show that, even if a large uterine weight is associated with increased uterine morcellation requirement and operative duration, a laparoscopic approach is safe and does not increase operative complications nor pain and/or length of hospital stay in women undergoing hysterectomy for a benign indication.

## Precis

In women with a large uterus undergoing hysterectomy for a benign indication, laparoscopic approach is safe and does not increase operative complications nor pain and/or length of hospital stay.

## Introduction

Hysterectomy is one of the most common surgical procedure in gynecology with three different surgical approaches (open, vaginal, and laparoscopic) (1). The laparoscopic approach was first performed in January 1988 by H Reich and is now the most common approach to minimally-invasive hysterectomy (2). In Switzerland, even if there are regional variations of surgical approach to hysterectomy for a benign indication, vaginal and abdominal hysterectomy are increasingly being replaced by laparoscopic hysterectomy (3).

Laparoscopic surgery is associated with faster recovery and decreased morbidity and mortality in comparison with an open approach (4). However, large uterine sizes can limit visualization during laparoscopy which can increase complications such as hemorrhage, organ & visceral injury, and may prolong operative duration (5). These complications may be increased if uterine morcellation is required. In addition, if uterine morcellation is done outside an endoscopic bag, complications such as retained or disseminated parasitic tissue have been documented in the medical literature as well as carcinoma cell spillage in case of unexpected malignancy (6). Even though minimally invasive approaches should be preferred over abdominal hysterectomy, due to their well-documented advantages, the American Congress of Obstetricians and Gynecologists does not necessarily recommend to perform laparoscopic hysterectomy in case of a large uterus (7).

Uterine size is usually estimated during the medical visit by a bimanual preoperative examination and/or a volumetric measure by ultrasound. It has been found that uterine size estimation by physicians, either by bimanual examination and/or ultrasound, has a strong, positive, and linear association with the actual pelvic specimen weight (8). In case of large uterine volumes and/or numerous leiomyomas of great size, ultrasound is less performing and it is recommended to use MRI to estimate uterine volume (9).

The main objective of this study was to evaluate if there was an association in between uterine weight and operative complications in women undergoing laparoscopic hysterectomy for a benign indication. Operative outcomes (need for uterine morcellation, operative duration, estimated blood loss), postoperative pain and hospital length of stay were also compared.

## Materials and Methods

Hundred and seventy women undergoing laparoscopic hysterectomy for a benign indication, with or without oophorectomy, in the Department of Pediatrics, Gynecology and Obstetrics of the Geneva University Hospitals from September 2015 to January 2020 were included in a randomized trial evaluating a fast-track (FT) protocol. The main objective of this trial was to compare the effectiveness of a FT protocol in laparoscopic hysterectomy for benign indications versus usual care in terms of costs, hospital length of stay, postoperative morbidity, and patient satisfaction.

Exclusion criteria were (1) the requirement for an additional surgical procedure, such as prolapse repair or urinary incontinence, and (2) the inability to speak French because the patients were required to complete a data collection logbook in French. All patients undergoing laparoscopic hysterectomy for a benign indication, with or without oophorectomy, who did not meet the exclusion criteria and who consented to participate in the study were included in the randomized trial. The study protocol was approved by the Cantonal Ethics Committee of Geneva (CCER 15–103).

We analyzed the database of this randomized trial. Women were divided in two groups according to the uterine weight, measured after the operation: small uterus (S-uterus) group (<250 grams) and large uterus (L-uterus) group (≥250 grams). The cut-off of 250 g was based on a systemic review by Driessen et al. who showed that uterine weight ≥250 g was associated with longer operative time and higher blood loss in case of laparoscopic hysterectomy (10).

The surgical procedure consisted of a standardized total laparoscopic hysterectomy. The women were placed in the lithotomy position with the arms alongside the body. A urinary Foley catheter was inserted into the bladder at the beginning of the procedure. Pneumoperitoneum was created using a Veress needle. The intra-abdominal pressure was of 12 mmHg during the procedure. One umbilical or supra-umbilical port (depending on the uterus size) was inserted for the optic laparoscope (5- or 10-mm diameter depending on the surgeon's preference), and three accessory ports were used for the standard laparoscopic instruments (5-mm diameter). A uterine manipulator (HOHL; Karl Storz, Tuttlingen, Germany) was used to mobilize the uterus during the dissection. The surgical steps were standardized according to the European Society for Gynecological Endoscopy (11). When the surgeon was unable to retrieve a large uterus transvaginally in one piece, morcellation techniques were used preferably using transvaginal cold morcellation to transabdominal power morcellation. The vaginal cuff was sutured with multifilament absorbable sutures using the intracorporeal knots technique. All the surgical procedures were performed by senior surgeons who are engaged in a regular surgical activity.

All women received a logbook to evaluate pain based on a visual analog scale (VAS) ranging from 0 (no pain) to 10 (worst possible pain).

Standard criteria for hospital discharge were applied: normal physical examination, no fever, effective non-opioid oral analgesia, normal feeding, absence of postoperative nausea and vomiting, independent mobility, and patient consent. Planned outpatient postoperative follow-up examinations were performed on days 7 and 30.

The primary outcome of this study was operative complications. The secondary outcomes were operative outcomes (need for uterine morcellation, operative duration, estimated blood loss), postoperative pain, and hospital length of stay.

Patient's characteristics (age, origin, body mass index (BMI), comorbidities, number of previous abdominal surgery and surgical indications) were collected from the previous existing database. Uterine weight was retrospectively retrieved from the pathologist report. Operative complications and morbidity during the first postoperative month were prospectively collected from the database of the Accident and Emergency (A&E) Department. Operative outcomes (need for uterine morcellation, operative duration, estimated blood loss) were retrospectively retrieved from the operative report and uterine morcellation was confirmed by the pathologic report. Postoperative pain was collected from the previous existing database and hospital length of stay was prospectively retrieved from the computerized record.

Categorical characteristics and outcomes were compared between the groups, and the statistical significance of differences were tested using the Fisher's exact test. For continuous variables, the differences were tested using a Student *T* test. Analyses were conducted using Stata and R software, and a *p* < 0.05 was considered as statistically significant.

## Results

In total, our secondary analysis included 159 patients. From the initial database of 170 patients, 10 patients were excluded from the randomized trial: two withdrew their consent, two decided not to undergo surgery, one underwent uterine artery embolization instead of surgery, four had an abdominal hysterectomy and one had a laparoscopic-assisted vaginal hysterectomy; and one patient, included in the randomized trial, was excluded from the secondary analysis because the pathologist report did not record the uterine weight.

Patients in both groups were similar in terms of age, body mass index, active smoking, comorbidities, and number of previous abdominal surgery. Previous abdominal surgeries included mainly cesarean section and laparoscopy for benign indications such as ovarian cyst, ectopic pregnancy, appendicitis, and cholecystitis. There were, however, differences in terms of surgical indication (Table 1). In terms of surgical indication, the S-uterus group comprised more patients with endometriosis/adenomyosis (36%) and the L-uterus group more patients with leiomyoma (93%).

**Table 1:** Patients' characteristics.

**Variable**	**S-uterus group (*n* = 92)**	**L-uterus group (*n* = 67)**	*p*-value^*^
Age, years	46 ± 7	47 ± 5	0.83
**Origin**			<0.01
Europe	68 (73.9%)	33 (49.2%)	
Africa	4 (4.3%)	16 (23.9%)	
Asia	2 (2.2%)	1 (1.5%)	
South America	18 (19.6%)	15 (22.4%)	
North America	0 (0%)	1 (1.5%)	
Oceania	0 (0%)	1 (1.5%)	
**BMI, kg/m** ^**2**^			0.99
Normal (18.5–24.9)	40 (43.5%)	29 (43.3%)	
Overweight (25.0–29.9)	28 (30.4%)	21 (31.3%)	
Obese (≥30.0)	24 (26.1%)	17 (25.4%)	
**Active smoking**	32 (34.8%)	13 (19.4%)	0.05
**Comorbidities**			
Diabetes	5 (5.4%)	1 (1.5%)	0.39
Hypertension	13 (14.1%)	8 (14.9%)	0.88
Hypercholesterolemia	9 (9.8%)	9 (13.4%)	0.64
Thromboembolic disease	7 (7.6%)	2 (3.0%)	0.37
**Previous abdominal surgery (number)**			0.21
0	35 (38.0%)	34 (50.7%)	
1	23 (25.0%)	16 (23.9%)	
≥2	34 (37.0%)	17 (25.4%)	
**Surgical indication**			<0.01
Leiomyoma	52 (56.5%)	62 (92.5%)	
Endometriosis/adenomyosis	33 (35.9%)	5 (7.5%)	
Cervical dysplasia	5 (5.4%)	0 (0%)	
Other (complex endometrial hyperplasia and prophylactic for a Li-Fraumeni syndrome)	2 (2.2%)	0 (0%)	

*Data are presented as mean ± standard deviation or n (%)*.

*S-uterus, small-uterus (<250 gr); L-uterus, large-uterus (>=250 gr); BMI, body mass index*.

* *p-values were calculated with the Fisher exact test, except for age tested with the Student T test*.

The mean uterine weight in the S-uterus group was 145 grams ± 53 grams and 458 grams ± 196 grams in the L-uterus group (*p* ≤ 0.01) (Table 2). Forty-one patients in the L-uterus group required uterine morcellation whereas only nine patients in the S-uterus group required uterine morcellation (p ≤ 0.01). Out of all the operations requiring morcellation, transvaginal morcellation was used in 84% of cases and abdominal power morcellation was used in 16% of cases.

**Table 2:** Operative characteristics.

**Variable**	**S-uterus group (*n* = 92)**	**L-uterus group (*n* = 67)**	*p*-value^*^
**Perioperative characteristics**			
Mean and range of uterine weight (grams)	145 (34–246) ± 53	458 (250–1,300) ± 196	<0.01
Uterine morcellation	9 (9.8%)	41 (61.2%)	<0.01
Mean total operative duration (min)	150 ± 55	176 ± 58	<0.01
Mean estimated blood loss (mL)	134 ± 101	163 ± 129	0.13
**Postoperative characteristics**			
VAS pain score on surgery day	5 ± 3	5 ± 3	0.42
VAS pain score on the day 1	4 ± 3	3 ± 2	0.03
Hospital stay (h)	62 ± 37	54 ± 21	0.10

*Data are presented as mean ± standard deviation or n (%)*.

*S-uterus, small-uterus; L-uterus, large-uterus; VAS, visual analog scale (0–10)*.

* *p-values were calculated with the Student T test, except uterine morcellation tested with the Fisher exact test*.

The mean total operative duration was 150 ± 55 min in the S-uterus group in comparison to 176 ± 58 min in the L-uterus group (p ≤ 0.01), corresponding to a significative increased operative duration of 17% in the large uterus group. The mean estimated blood loss was 134 ± 101 mL in the S-uterus group and 163 ± 129 mL in the L-uterus group (*p* = 0.13).

The mean VAS pain score on the day of surgery was 5 ± 3 in both groups. On day 1 postoperatively, the mean VAS pain score was 4 ± 3 in the S-uterus group and 3 ± 2 in the L-uterus group (*p* = 0.03).

One perioperative complication was recorded in the S-uterus group. A woman with two prior cesarean sections had a bladder perforation during dissection. It was diagnosed during surgery and surgically repaired during the same operation. The patient went home on postoperative day 3 with a Foley catheter that was left in the bladder for a total of 7 days. After removal of the Foley catheter, the patient did not suffer any urinary sequelae.

We recorded 38 postoperative complications in the S-uterus group (41%) and 25 in the L-uterus group (37%, *p* = 0.73). The frequency and severity of complications were similar between the two groups according to the Clavien–Dindo classification (Table 3). Only one complication required surgical revision. It was the case of a woman with a 224 grams uterus in the S-uterus group. The uterus was vaginally morcelated with an iatrogenic 2 cm tear of the vagina. The patient presented with important postoperative bleeding on day 2 needing surgical suture. The rest of the postoperative period was uneventful and the follow-up examination on day 30 showed a total healing of the vagina with no sequelae.

**Table 3:** Details of complications according to Clavien–Dindo classification.

	**S-uterus group (*n* = 92)**	**L-uterus group (*n* = 67)**	*p*-value^*^
Any complication	38 (41.3%)	25 (37.3%)	0.73
Grade 1	7 (7.6%)	8 (11.9%)	0.51
Important post-operative pain	1	0	
Scab fall	1	1	
Vaginal hematoma	2	2	
Fever of unknown origin	0	1	
Urinary retention	2	4	
Bladder perforation repaired during initial surgery	1	0	
Grade 2	30 (32.6%)	17 (25.4%)	0.42
Anemia	3	5	
Constipation	4	1	
Urinary tract infection	11	5	
Vaginal infection	6	5	
Vaginal hematoma infection	1	1	
Vaginal cuff abscess	1	0	
Vaginal cuff dehiscence	2	0	
Pulmonary embolism	1	0	
Left pelvic plexus thrombus	1	0	
Grade 3	1 (1.1%)	0 (0.0%)	
Vaginal tear requiring surgery	1	0	
Grade 4	0	0	

*Data are presented as n (%) or n*.

*S-uterus, small-uterus; L-uterus, large-uterus*.

* *p-values were calculated with the Fisher exact test*.

The mean hospital stay was 62 ± 37 hours in the S-uterus group and 54 ± 21 hours in the L-uterus group (*p* = 0.10).

## Discussion

To our knowledge, this is one of the first studies investigating the risk associated in between uterine weight and operative complications in women undergoing laparoscopic hysterectomy for a benign indication. Our study shows that a laparoscopic approach is safe and does not increase operative complications nor pain and/or length of hospital stay in women with large uterine sizes undergoing laparoscopic hysterectomy for a benign indication.

A study by Louis et al. evaluating the association between uterine weight and post-hysterectomy complications across vaginal, laparoscopic, and abdominal approaches showed that, even though uterine weight was an independent risk factor for post-hysterectomy complications, uterine weight alone was not an appropriate indication for abdominal hysterectomy (12). Our study confirms these findings, where no perioperative complications were found in the L-uterus group and postoperative complications were similar between the S-uterus group and the L-uterus group.

Final guidance of the U.S. Food and Drug Administration (FDA) recommends limiting the use of power laparoscopic morcellation because of the risk of an unexpected malignancy, such as uterine sarcoma, reported as high as 1 in 350 (0.29%) and, if disseminated, having a worse progression-free survival and overall survival (13, 14). For laparoscopic hysterectomy, recent studies have shown that the use of a specially designed endoscopic bag could be the solution as it is a simple, fast, and a safe technique for containing tissue during electromechanical morcellation (15). Currently, the American College of Obstetrics and Gynecology, the Society of Gynecologic Oncology, and the American Association of Gynecologist Laparoscopists all suggest bags as potential solutions to prevent dissemination of tissue (16). If transvaginal morcellation is necessary, it should also be done in a morcellation bag, using vaginal retractors to protect the genitourinary tract under visual control (17). Our study showed that as of a uterine weight of 800 grams, morcellation was required in 100% of cases. Thus, if a large uterus is preoperatively suspected with a risk of morcellation, a pre-operative screening including cervical cytology, endometrial biopsy, pelvic ultrasound and/or MRI should be performed, and the patient should be informed about the risks (18). In our institution, if morcellation is necessary, we favor transvaginal morcellation as it reduces incision-related morbidity. If the patient presents a narrow vagina, we use abdominal power morcellation after obtaining patient's specific consent.

Operative time was increased by 17% in the L-uterus group, mainly due to uterine morcellation, with an additional 26 min required in the L-uterus group in comparison to the S-uterus group. This finding is in agreement with a prospective study by Maccio et al. where surgical data were stratified by uterine weight in four groups and showed that increased uterine weight was associated with longer operative time (19). This downside is largely offset by the advantages of laparoscopic surgery. Indeed, laparoscopic hysterectomy is associated with decreased hospital length of stay and faster recovery time compared with laparotomy (20).

An interesting finding in our study was the significant reduced mean VAS pain score on day 1 postoperative and the reduced mean hospital stay in the L-uterus group. Our results showed that the S-uterus group comprised more patients with endometriosis/adenomyosis (36%) and the L-uterus group more patients with leiomyoma (93%), which was not surprising as the main cause of an increased uterus size is the presence of leiomyoma. The fact that the S-uterus group comprised more patients with endometriosis/adenomyosis could explain why their mean VAS pain score on day 1 postoperative and their mean hospital stay were increased in comparison to the L-uterus group. Indeed, a study by Aredo et al. showed that even after optimized surgery treatment for endometriosis, central sensitization of the nervous system and myofascial dysfunction can continue to generate pain from myofascial trigger points (21). This finding is supported by Wong et al. who showed that chronic endometriosis was strongly correlated with postoperative opioid use (22).

Currently, the American Congress of Obstetricians and Gynecologists encourage minimally invasive surgery which includes laparoscopic and vaginal hysterectomy, but some authors argue that the vaginal route should be preferred because it is associated with shorter operative time, lower risk of vaginal dehiscence & conversion to laparotomy, and lower costs (7, 23). Nevertheless, no study has compared a laparoscopic to a vaginal surgical approach in terms of operative complications, stratifying for uterine weight.

The major limitation of our study is that it is a secondary analysis of a randomized trial, with data collected prospectively and retrospectively, evaluating only one surgical approach. A randomized trial including women with a large uterus to evaluate the risk of operative complications with the laparoscopic and vaginal approach should be undertaken.

The results from this secondary analysis of a randomized trial show that even if large uterine weight is associated with increased uterine morcellation requirement and operative duration, a laparoscopic approach is safe and does not increase operative complications nor pain and/or hospital length of stay in women undergoing hysterectomy for a benign indication.

## Data Availability Statement

The raw data supporting the conclusions of this article will be made available by the authors, without undue reservation.

## Ethics Statement

The studies involving human participants were reviewed and approved by Cantonal Ethics Committee of Geneva (CCER 15-103). The patients/participants provided their written informed consent to participate in this study.

## Author Contributions

SL: conception, statistical analysis, and manuscript preparation. MB: statistical analysis and manuscript revision. PP: manuscript revision. JD: conception and manuscript revision. All authors listed have made a substantial, direct and intellectual contribution to the work, and approved it for publication.

## Conflict of Interest

The authors declare that the research was conducted in the absence of any commercial or financial relationships that could be construed as a potential conflict of interest.

## Publisher's Note

All claims expressed in this article are solely those of the authors and do not necessarily represent those of their affiliated organizations, or those of the publisher, the editors and the reviewers. Any product that may be evaluated in this article, or claim that may be made by its manufacturer, is not guaranteed or endorsed by the publisher.
